# Interferon regulatory factor 4 attenuates Notch signaling to suppress the development of chronic lymphocytic leukemia

**DOI:** 10.18632/oncotarget.9596

**Published:** 2016-05-25

**Authors:** Vipul Shukla, Ashima Shukla, Shantaram S. Joshi, Runqing Lu

**Affiliations:** ^1^ Department of Genetics Cell Biology and Anatomy, University of Nebraska Medical Center, Omaha, NE, USA

**Keywords:** IRF4, Notch signaling, CLL, B1 cells

## Abstract

Molecular pathogenesis of Chronic Lymphocytic Leukemia (CLL) is not fully elucidated. Genome wide association studies have linked Interferon Regulatory Factor 4 (IRF4) to the development of CLL. We recently established a causal relationship between low levels of IRF4 and development of CLL. However, the molecular mechanism through which IRF4 suppresses CLL development remains unclear. Deregulation of Notch signaling pathway has been identified as one of the most recurrent molecular anomalies in the pathogenesis of CLL. Yet, the role of Notch signaling as well as its regulation during CLL development remains poorly understood. Previously, we demonstrated that IRF4 deficient mice expressing immunoglobulin heavy chain Vh11 (IRF4^−/−^Vh11) developed spontaneous CLL with complete penetrance. In this study, we show that elevated Notch2 expression and the resulting hyperactivation of Notch signaling are common features of IRF4^−/−^Vh11 CLL cells. Our studies further reveal that Notch signaling is indispensable for CLL development in the IRF4^−/−^Vh11 mice. Moreover, we identify E3 ubiquitin ligase Nedd4, which targets Notch for degradation, as a direct target of IRF4 in CLL cells and their precursors. Collectively, our studies provide the first *in vivo* evidence for an essential role of Notch signaling in the development of CLL and establish IRF4 as a critical regulator of Notch signaling during CLL development.

## INTRODUCTION

Chronic Lymphocytic Leukemia (CLL) is a clinically heterogeneous B cell malignancy. Despite considerable progress in our current understanding of CLL, the molecular events underlying the complex pathogenesis of CLL have not been fully elucidated. Recent Whole Genome Sequencing studies have identified mutational activation of Notch signaling pathway as one of the most recurrent molecular events in human CLL [1–5]. Moreover, the CLL patients carrying mutations in Notch signaling pathway have poor clinical outcomes and an increased tendency towards Richter transformation to Diffused Large B cell Lymphoma [1, 2, 6]. Notch signaling is an evolutionarily conserved pathway that regulates a myriad of cellular processes [7]. In CLL patients, Notch signaling pathway can be activated by mutations that primarily affect the stability of Notch1 protein [2, 5]. Notch mutations in CLL patients cause frameshift deletions leading to generation of protein without the PEST domain, that is critical for degradation of Notch proteins [2, 5, 7]. Other than the mutational activation, studies have also reported constitutively high expression of Notch1 and Notch2 leading to activation of Notch signaling in human CLL cells [8]. *In vitro* studies have also provided evidence for a role of Notch signaling in promoting the survival and chemo-resistance of CLL cells [9, 10]. Although, these studies have linked aberrant Notch signaling to the pathogenesis of CLL *in vitro*, whether Notch signaling is critical for CLL development *in vivo* remains unknown. Furthermore, the molecular pathways that lead to the deregulated Notch signaling in CLL cases without Notch mutations are still poorly defined.

Interferon Regulatory Factor 4 (IRF4) belongs to the IRF superfamily of transcription factors and regulates multiple developmental stages and functional processes in B lymphocytes [11, 12]. In distinct B cell malignancies, IRF4 has been shown to possess both tumor suppressive and pro-oncogenic functions [11, 12]. Recent studies from our group and others have established an important role of IRF4 in the development of CLL [13–16]. Genome Wide Association (GWA) study linked single nucleotide polymorphisms (SNPs) in the 3′ untranslated region of *irf4* gene locus present in majority of CLL patients (86%) to the development of CLL [13, 16]. Using distinct mouse models we have recently established a causal link between low levels of IRF4 and CLL development [14, 15]. Vh11 knock-in (KI) mouse is a genetically engineered mouse which expresses a prearranged immunoglobulin heavy chain gene family Vh11. B cells expressing Vh11 heavy chain predominantly develops into a specialized B cell subset known as B1 cells that are also the presumed precursors of CLL cells in rodents [17]. Remarkably, our studies revealed that IRF4 deficient Vh11 KI (IRF4^−/−^Vh11) mice developed spontaneous CLL at complete penetrance [15]. Interestingly, we also showed that low levels of IRF4 dramatically accelerates CLL development in a spontaneous, late-onset; New Zealand Black mouse model of CLL [14, 18]. Although our studies have established a causal relationship between low levels of IRF4 and CLL development, the molecular mechanism through which IRF4 suppresses CLL development remains unknown.

Interestingly, a recent study described expansion of a specialized mature B cell subset known as Marginal Zone B cells (MZ B cells) in IRF4 deficient mice that was attributed to higher levels of Notch2 receptor and associated Notch signaling [19]. Although the precise mechanism through which IRF4 regulates Notch signaling remains unclear, this study identified IRF4 as a potential novel regulator of Notch signaling in mature B cells. Given the possible connection between Notch signaling and CLL development, we hypothesized that in the IRF4^−/−^Vh11 mice Notch signaling is also deregulated and the deregulation plays a critical role in CLL development. IRF4^−/−^Vh11 mouse is regarded as a novel mouse CLL model because it mimics a predominant genetic predisposition to CLL [20]. Therefore, IRF4^−/−^Vh11 mice are very useful in understanding not only the molecular mechanism through which IRF4 controls CLL development but also the pathogenesis of CLL in general. In the present studies we examined the role of Notch signaling and its regulation by IRF4 in the development of CLL in IRF4−/−Vh11 mice as well as in human CLL cells.

## RESULTS

### IRF4^−/−^Vh11 CLL cells display hyperactive Notch signaling

**Figure 1:**
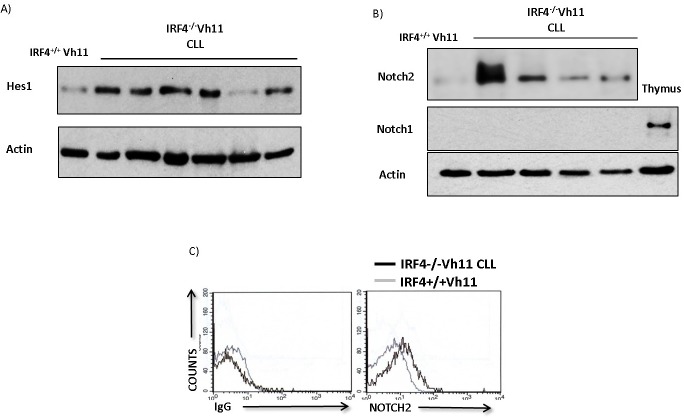
IRF4^**−/−**^Vh11 CLL cells display hyperactive Notch signaling and express high levels of Notch2 receptor **A.** Western-blot analysis to detect the levels of Hes1 protein in IRF4^−/−^Vh11 CLL cells compared to IRF4^+/+^Vh11 B cells isolated from spleen. Each lane represents CLL cells from an IRF4^−/−^Vh11 mouse. **B.** Western-blot to detect the levels of Notch2 and Notch1 proteins in IRF4^−/−^Vh11 CLL cells. Each lane represents an individual CLL sample. Thymus is used as a positive control for Notch1 protein and actin is used as loading control. **C.** Histograms showing Notch2 cell surface staining in IRF4^−/−^Vh11 CLL cells compared to IRF4^+/+^Vh11 B cells as detected by Flow cytometry. Left panel shows isotype control antibody (IgG) staining and right panel shows Notch2 staining. Gray line represents gating on IRF4^+/+^Vh11 B cells and black line indicates IRF4^−/−^Vh11 CLL cells. The data shown is representative of at least three independent experiments.

We hypothesized that Notch signaling plays a critical role in the development of CLL in IRF4^−/−^Vh11 mice. To study the activation state of Notch signaling we measured the levels of canonical Notch target gene, Hes1 [9]. Hes1 has been previously shown to be upregulated in primary human CLL cells [8, 9]. Our preliminary analysis also showed upregulation of Hes1 mRNA in primary human CLL cells compared to normal human B cells (Supplementary Figure S1). Interestingly, using western-blot analysis we found Hes1 levels to be significantly upregulated in IRF4^−/−^Vh11 CLL cells compared to IRF4^+/+^Vh11 B cells (Figure 1A).

Notch protein family comprises of four different Notch paralogues from Notch1 through Notch4 therefore, we wanted to identify the predominant Notch paralogue(s) expressed in the IRF4^−/−^Vh11 CLL cells. Using western-blot analysis our studies revealed Notch2 protein as the predominant Notch paralogue expressed in IRF4^−/−^Vh11 CLL cells (Figure 1B). The expression levels of Notch1 (Figure 1B), Notch3 and Notch4 (data not shown) were barely detectable or undetectable in IRF4^−/−^Vh11 CLL cells. These findings are consistent with previous findings that described expression of Notch2 protein as the predominant Notch paralogue in mature murine B cells [21]. Furthermore, the levels of Notch2 protein detected in IRF4^−/−^Vh11 CLL cells were significantly higher compared to IRF4^+/+^Vh11 B cells (Figure 1B). Also, we detected activated form of Notch2 intracellular domain in IRF4^−/−^Vh11 CLL cells (Figure S2A). Additionally, we used a flow cytometry based assay that also showed a significant upregulation of Notch2 protein on cell surface of IRF4^−/−^Vh11 CLL cells (Figure 1C). Of note, consistent with a recent study, we did not observe a significant change in the Notch2 mRNA expression suggesting that high levels of Notch2 protein in IRF4^−/−^Vh11 CLL cells is likely a result of a post-transcriptional regulation [19]. Moreover, our results here show that upregulation of Notch2 protein and associated Notch signaling are common features of IRF4^−/−^Vh11 CLL cells. We further deleted Notch2 in B cells by breeding the CD19cre mouse to mouse carrying conditional alleles for *notch2* gene (CD19creNotch2^fl/fl^). B cells from CD19creNotch2^fl/fl^ mice showed efficient Notch2 deletion, accompanied with a dramatic downregulation of Hes1 (Figure S2B and S2C). These results indicate that Notch2 protein is the major contributor of Notch signaling in mature B cells and its loss leads to a profound abrogation of Notch signaling *in vivo*. Moreover, the loss of Notch2 expression apparently is not compensated for by other Notch protein family members as indicated by a strong decrease in Hes1 levels in B cells from CD19cre Notch2^fl/fl^ mice.

### Notch signaling promotes the survival and proliferation of B1 cells and CLL cells

**Figure 2:**
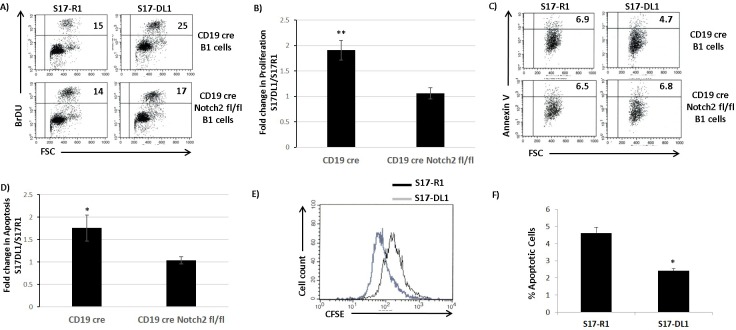
Notch signaling promotes the survival and proliferation of B1 cells and CLL cells **A.** Flow cytometry analysis showing the BrDU incorporation assay for cell proliferation of CD19cre control and CD19cre Notch2^fl/fl^ B1 cells co-cultured with S17-R1 and S17-DL1 stromal-cells for 48 hours. The numbers in the upper right quadrant of each dot plot represents the percentage of BrDU positive cells. **B.** Bar graphs showing the statistical analysis of BrDU incorporation assay from three independent experiments. The data is represented as fold change in proliferation observed on S17-DL1 stromal-cells compared to S17-R1 control stromal-cells. **C.** Flow cytometry analysis showing Annexin V staining to detect apoptotic cells among CD19cre and CD19cre Notch2^fl/fl^ B1 cells cultured with S17-R1 and S17-DL-1 stromal-cells for 48 hours. The numbers in each dot plot represents the percentage of Annexin V positive cells in the upper right quadrant. **D.** Bar graph showing the statistical analysis of Annexin V staining of CD19cre and CD19cre Notch2^fl/fl^ B1 cells from five independent experiments. The data is represented as fold change in proliferation observed on S17-DL1 stromal-cells compared to S17-R1 control stromal-cells. **E.** Histograms representing CFSE dye dilution experiment to measure proliferation of IRF4^−/−^Vh11 CLL cells co-cultured with S17-R1 (black line) and S17-DL1 (gray line) stromal-cells. Black line represents CLL cells cultured on S17-R1 stromal-cells and gray line represents. **F.** Bar graphs showing the percentages of Annexin V positive IRF4^−/−^Vh11 CLL cells co-cultured with S17-R1 and S17-DL1 stromal-cells from three independent experiments. *p value ≤0.01. **p value ≤0.05.

We wanted to determine the effect of Notch signaling on survival and proliferation of B1 cells (CLL precursors) and CLL cells. Briefly, we expressed the Notch ligand, Delta like 1 (S17-DL1) in S17 stromal-cells to trigger Notch signaling. S17 cells expressing empty vector were used as controls (S17-R1). B1 cells cultured on S17-DL1 stromal-cells showed strong activation of Notch signaling, as measured by Hes1 protein induction (Figure S3). We then isolated B1 cells from the peritoneal cavities of CD19creNotch2^+/+^ and CD19creNotch2^fl/fl^ mice and cultured them on control or Notch ligand expressing stromal-cells. Interestingly, the CD19creNotch2^+/+^ B1 cells cultured on S17-DL1 stromal-cells proliferated significantly faster compared to the cells cultured on S17-R1 stromal-cells as measured by BrdU incorporation assay (Figure 2A and 2B). Importantly, the increase in proliferation observed on wild type B1 cells was mostly abolished when Notch2 was deleted in B1 cells (CD19creNotch2^fl/fl^) (Figure 2A and 2B). Similarly, we also observed a decrease in apoptosis of CD19creNotch2^+/+^ B1 cells, cultured on S17-DL1 stromal-cells (Figure 2C and 2D). The increase in survival observed on S17-DL1 stromal-cells was again negated in B1 cells isolated from CD19creNotch2^fl/fl^ mice (Figure 2C and 2D).

We next examined the effect of Notch signaling on CLL cells derived from IRF4^−/−^Vh11 mice. CFSE dilution assay revealed that IRF4^−/−^Vh11 CLL cells cultured on S17-DL1 stromal-cells proliferated much faster than their counterparts cultured on S17-R1 stromal-cells (Figure 2E). Also, the survival of IRF4^−/−^Vh11 CLL cells was enhanced when cultured on Notch ligand expressing (S17-DL1) stromal-cells (Figure 2F). In summary, these results demonstrate that CLL cells and their precursors are responsive to Notch signaling, which promotes their survival and proliferation.

### Notch2 in critical for CLL development in IRF4^−/−^Vh11 mice

**Figure 3:**
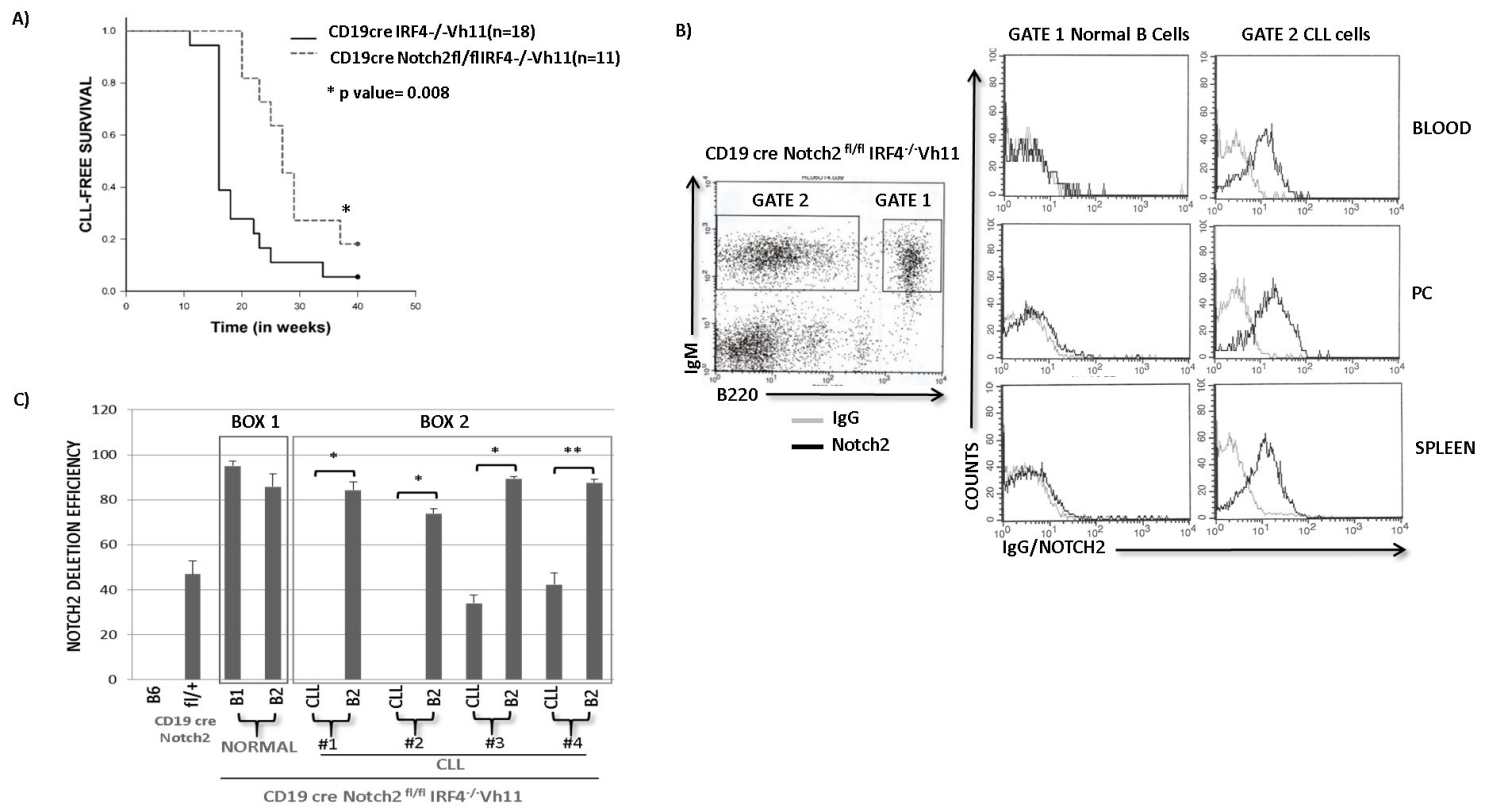
Notch2 receptor is critical for CLL development in IRF4^**−/−**^Vh11 mice **A.** Kaplan Meier Survival analysis (log-rank test) for CLL development in CD19creNotch2^fl/fl^IRF4^−/−^Vh11 mice (*n* = 11) (dashed line) compared to CD19creIRF4^−/−^Vh11 mice (*n* = 18) (solid line). Blood was analyzed biweekly to monitor CLL development that is considered as an event represented on Y-axis. X-axis represents time in weeks. **B.** Left panel shows flow cytometry staining of IgM and B220 in CD19creNotch2^fl/fl^IRF4^−/−^Vh11 mice. Normal untransformed B cells are IgM+ and B220 high (Gate 1) and CLL cells are IgM+ and B220 medium/dim (Gate 2). Right panel shows histograms representing IgG (gray line) or Notch2 (black line) staining in Normal B cells and CLL cells from Blood, Peritoneal Cavity (PC) and Spleen. **C.** Bar graph showing qRT-PCR data representing absolute Notch2 deletion efficiencies. A deletion efficiency of 47 as observed in CD19creNotch2^fl/fl^ B cells signifies 47% *notch2* gene deletion. Box1 contains B1 (CLL precursors) and B2 (normal B) cells from CD19cre Notch2^fl/fl^IRF4^−/−^Vh11 mice without overt signs of CLL. Box2 encloses Notch2 deletion efficiencies in CLL and B2 cells from four different CD19creNotch2^fl/fl^IRF4^−/−^Vh11 mice with overt CLL. *p value ≤0.001 **p value ≤0.01.

We next wanted to determine the role of Notch signaling in the development of CLL in IRF4^−/−^Vh11 mice. To address this goal we utilized a genetic approach to delete Notch2 gene in the IRF4^−/−^Vh11 mice. Briefly, we bred the IRF4^−/−^Vh11 mice with the CD19creNotch2^fl/fl^ mice to generate CD19creNotch2^fl/fl^IRF4^−/−^Vh11 mice. Blood was analyzed biweekly from CD19creNotch2^fl/fl^ IRF4^−/−^Vh11 mice to monitor the emergence of CLL cells and CD19creIRF4^−/−^Vh11 mice were also analyzed as control. Interestingly, compared to CD19creIRF4^−/−^Vh11 mice (*n* = 18) we observed a significant delay in the onset of CLL development in CD19creNotch2^fl/fl^ IRF4^−/−^Vh11 mice (*n* = 11) (Figure 3A). The disease latency increased from 19.5 weeks in CD19creIRF4^−/−^Vh11 mice to 28.8 weeks in CD19creNotch2^fl/fl^ IRF4^−/−^Vh11 mice (Figure 3A). Surprisingly, upon further analysis we observed that the CLL cells which emerged from CD19creNotch2^fl/fl^IRF4^−/−^Vh11 mice, continued to express high levels of Notch2 protein on their cell surface (Figure 3B right panel). In total, we analyzed 15 mice with Notch2^fl/fl^ IRF4^−/−^Vh11 genotype and all of them eventually showed emergence of CLL cells which retained Notch2 expression on their cell surface. These results can have three plausible explanations. Firstly, these findings can be caused by insufficient CD19cre mediated Notch2 deletion in B cells of the IRF4^−/−^Vh11 mice. Secondly, these findings can also be caused by a defect in B cell development upon Notch2 deletion that prevents the generation of CLL precursors (B1 cells) in the IRF4^−/−^Vh11 mice. Thirdly, these findings can be explained by our hypothesis which implies that Notch2 is critical for CLL development and without it, CLL cells cannot be generated.

To distinguish between these different scenarios, we analyzed Notch2 expression in CD19creNotch2^fl/fl^IRF4^−/−^Vh11 mice that were still at the early stages of CLL development. This allowed us to simultaneously evaluate a CLL population as well as a detectable population of untransformed normal B cells (B2 cells) in the same mice. Intriguingly, our analysis revealed that only the CLL cells from CD19creNotch2^fl/fl^IRF4^−/−^Vh11 mice expressed high levels of Notch2 protein while, the normal B cells from the same mice displayed very low to undetectable levels of Notch2 protein (Figure 3B right panel). These findings were consistent in cells isolated from several tissues including peritoneal cavity (PC), spleen and blood (Figure 3B). Concurrently, we devised a real-time PCR based assay to precisely calculate the efficiency of Notch2 deletion among different cell populations. We specifically designed PCR primers in region within the Notch2 conditional allele that is flanked by the loxP sites. This approach allows for PCR amplification from Notch2 alleles that have not undergone cre mediated deletion. Furthermore, we also amplified a non-related region in the genome and used it as control to normalize the result. This method precisely calculates absolute deletion efficiencies for the *notch2* alleles. To validate this method, we extracted genomic DNA from B cells of wildtype B6 and CD19creNotch2^fl/+^ mice. As expected, the assay revealed a Notch2 deletion efficiency of 47% in B cells isolated from CD19creNotch2^fl/+^ heterozygous mice compared to wildtype B cells (Figure 3C). Using this assay we first wanted to rule out the possibility for any aberrant B cell developmental defect in CD19creNotch2^fl/fl^IRF4^−/−^Vh11 mice. To this end, we analyzed CD19creNotch2^fl/fl^IRF4^−/−^Vh11 mice of 2-3 months of age with no overt signs of CLL. Flow cytometry analysis showed efficient generation of CD5+IgM+ B1 cells in the peritoneal cavities of CD19creNotch2^fl/fl^IRF4^−/−^Vh11 mice at a frequency that is comparable to that of IRF4^+/+^Vh11 and IRF4^−/−^Vh11 mice (Figure S4). Furthermore, the B1 and B2 (normal B cells) cells isolated from CD19creNotch2^fl/fl^IRF4^−/−^Vh11 mice with no CLL displayed very high efficiencies of *notch2* gene deletion (~90%) (Figure 3C Box1). These results indicate that Notch2 is not essential for B1 cell generation in the CD19creNotch2^fl/fl^ IRF4^−/−^Vh11 mice. Together, our results rule out the first explanation by demonstrating that *notch2* gene is efficiently deleted in all B cell subsets in the CD19creNotch2^fl/fl^IRF4^−/−^Vh11 mice. Additionally, our results also discredit the second explanation by showing that Notch2 is dispensable for the generation of B1 cells in the IRF4^−/−^Vh11 mice.

We next used FACS to sort CLL cells and normal B cells (B2 cells) from CD19creNotch2^fl/fl^IRF4^−/−^Vh11 mice and extracted genomic DNAs from sorted cells for analyzing the respective *notch2* deletion efficiencies. The normal B cells (B2 cells) isolated from CD19creNotch2^fl/fl^IRF4^−/−^Vh11 displayed high efficiency of *notch2* deletion (≥ 90%) (Figure 3C Box2). Whereas, the CLL cells from the same mice displayed significantly lower *notch2* gene deletion efficiencies (Figure 3C Box2). It appears that CLL cells in some mice (mice 1 and 2) completely escaped *notch2* gene deletion (close to 0% deletion efficiency) while, in other mice (mice 3 and 4) the CLL cells showed 30-40% *notch2* gene deletion efficiencies (Figure 3C Box2). 30-40% deletion efficiency in these mice may reflect a mixed CLL population with heterozygous Notch2 deletion. However, it is worth pointing out that even in those mice, we did not observe a corresponding decrease in the Notch2 protein levels in the CLL populations (data not shown). In summary, our studies here show that Notch2 is indispensable for the generation of CLL in IRF4^−/−^Vh11 mice, indicating that Notch signaling is critical for CLL development in IRF4^−/−^Vh11 mice.

### IRF4 regulates the E3 ubiquitin ligase Nedd4 in IRF4^−/−^Vh11 CLL cells

**Figure 4:**
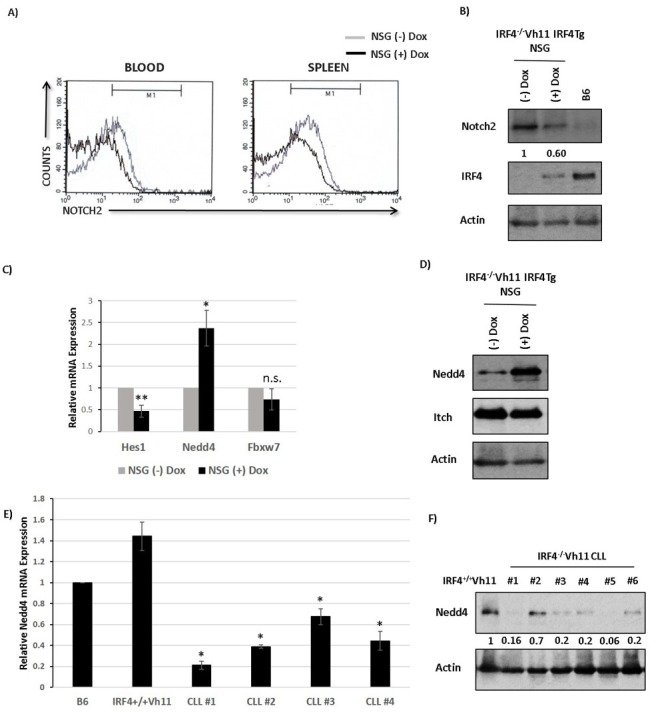
IRF4 regulates E3 ubiquitin ligase Nedd4 in CLL cells **A.** Histograms showing Notch2 staining in CLL cells isolated from blood and spleen of NSG mice fed with (black line) or without dox water for 3 weeks (gray line). **B.** Western-blot analysis to detect Notch and IRF4 levels in CLL cells isolated from NSG mice fed with or without dox water for 3 weeks. The numbers below represents normalized relative expression. B cells isolated from B6 mice are used as a measure of endogenous levels of IRF4. Actin is used as loading control. **C.** Bar graph representing the relative mRNA expression of Hes1, Nedd4 and Fbxw7 in CLL cells isolated from NSG mice fed with or without dox water for 3 weeks. **D.** Western-blot analysis to measure Nedd4 and Itch protein levels in NSG mice fed with or without dox. **E.** Bar graph showing the relative mRNA expression of Nedd4 in four different IRF4^−/−^Vh11 CLL samples compared to B cells isolated from wildtype B6 and IRF4^+/+^Vh11 mice. **F.** Western-blot analysis to measure the levels of Nedd4 protein in IRF4^−/−^Vh11 CLL samples compared to IRF4^+/+^Vh11 B cells. The numbers at the bottom represents Nedd4 expression measured by densitometric analysis using ImageJ software. Actin is used as the loading control. *p value ≤0.01 **p value ≤0.05.

Our results show that IRF4^−/−^Vh11 CLL cells express high levels of Notch2. However, how the expression levels of Notch2 are regulated by IRF4 remains unclear. To decipher the molecular mechanism, we reconstituted the expression of IRF4 in CLL cells by using a Doxycycline (Dox) inducible IRF4-transgenic model (IRF4^−/−^Vh1IRF4Tg) (Figure S5). The IRF4-deficient CLL cells carrying the IRF4-transgene were transplanted to NOD-scid gamma deficient (NSG) immunocompromised mice (Figure S5). IRF4-transgene was then induced in CLL cells by feeding the NSG mice with Dox containing water (NSG(+)Dox) while, mice fed with regular water were used as controls (NSG(−)Dox) (Figure S5). After three weeks of Dox treatment, we observed that IRF4 reconstitution led to a decrease in the cell surface levels of Notch2 receptor on CLL cells compared to NSG control mice in both blood and spleen (Figure 4A). We also observed a decrease in total levels of Notch2 protein in CLL cells upon IRF4 induction, as detected by western-blot analysis (Figure 4B) while, the mRNA expression of Notch2 remained unaffected (Figure S6). These results indicate that IRF4 downregulates expression of Notch2 and that the defect in Notch2 expression can be corrected upon IRF4 reconstitution in IRF4^−/−^Vh11 mice.

We further performed RNA sequencing (RNA-seq) of CLL cells isolated from mice treated with or without Dox to identify differentially expressed genes that could affect Notch protein turnover. A previous study linked reduced expression of an E3 ubiquitin ligase gene Fbxw7 to the increased Notch protein levels in the IRF4 deficient B cells [19]. However, Fbxw7 expression was not significantly affected upon IRF4 reconstitution (data not shown). Intriguingly, our RNA-seq data revealed an increase in expression of a different E3 ubiquitin ligase, Nedd4 upon IRF4 reconstitution (data not shown). Importantly, Nedd4 has been previously shown to ubiquitinate and degrade Notch receptors in drosophila and mammalian cellular systems [22–25]. We confirmed RNA-seq results RNA-seq results by real-time PCR (Figure 4C). Notably, reconstitution of IRF4 also led to a decrease in the expression of canonical Notch target gene Hes1 (Figure 4C). Western-blot analysis further showed a profound increase in Nedd4 protein expression upon IRF4 reconstitution whereas the levels of another E3 ubiquitin ligase, Itch which belongs to the same protein family as Nedd4, remained unchanged (Figure 4D). We further analyzed the mRNA and protein expression of Nedd4 in CLL cells. Compared to IRF4^+/+^Vh11 B cells, the mRNA and protein levels of Nedd4 were dramatically reduced in IRF4^−/−^Vh11 CLL cells (Figure 4E and 4F). Taken together, these studies identify Nedd4 as a potential IRF4 target gene and the major E3 ubiquitin ligase that is downregulated in the IRF4^−/−^Vh11 CLL cells.

### IRF4 directly binds to *nedd4* gene

**Figure 5:**
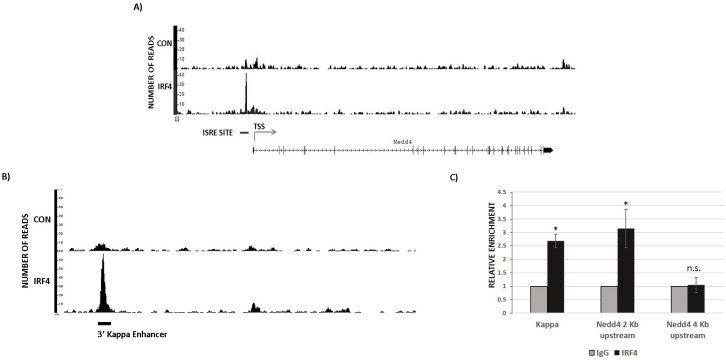
IRF4 directly binds to *nedd4* gene **A.** ChIP-seq data showing endogenous IRF4 binding at nedd4 gene locus in B1 cells isolated from IRF4^+/+^Vh11 mice. Immunoprecipitation of DNA fragments using anti-IRF4 antibody from IRF4^−/−^Vh11 B1 cells is used as control. TSS represents transcription start site and ISRE represents Interferon Stimulated Response Elements located in *nedd4* gene promoter. **B.** ChIP-seq data showing IRF4 binding to the 3′ kappa light chain enhancer used as positive control in IRF4^+/+^Vh11 B1 cells. **C.** Bar graph representing the data from conventional ChIP assay in B1 cells using IgG and anti IRF4 antibody. Kappa represents primers spanning the 3′ enhancer in the Kappa Ig light chain locus used as positive control for IRF4 binding. The data shown in **C.** is representative of three independent experiments. *p value ≤0.01.

We performed Chromatin Immunoprecipitation sequencing (ChIP-seq) to identify genome wide binding sites for IRF4 in B1 cells. For this study, IRF4^+/+^Vh11 B1 cells were used to map IRF4 binding sites and IRF4^−/−^Vh11 B1 cells were used as control cells. Strikingly, ChIP-seq revealed a robust binding of IRF4 in the promoter region of *nedd4* gene (Figure 5A). Furthermore, the IRF4 binding peak was mapped to a region harboring a canonical Interferon-Stimulated Response Element (ISRE) DNA motif (Figure 5A). The ISRE element was present 2 kilobases (kb) upstream to the transcription start site (TSS) in the *nedd4* gene promoter (Figure 5A). IRF4 has been previously shown to bind 3′ enhancer in the kappa immunoglobulin light chain locus [26]. Our ChIP-seq screen also showed a strong binding peak for IRF4 in the 3′ kappa enhancer region, ascertaining the specificity of our assay (Figure 5B). The IRF4 binding to the ISRE motif in the *nedd4* gene locus was further confirmed by the conventional ChIP assay, which showed significant enrichment of IRF4 binding at *nedd4* gene promoter in IRF4^+/+^Vh11 B1 cells (Figure 5C). Notably, we did not observe IRF4 binding at a region 4kb upstream to the TSS (Figure 5C). In summary, our results indicate that Nedd4 is a direct target of IRF4 in B1 cells.

### IRF4 regulates Nedd4 expression in B1 but not B2 cells

**Figure 6:**
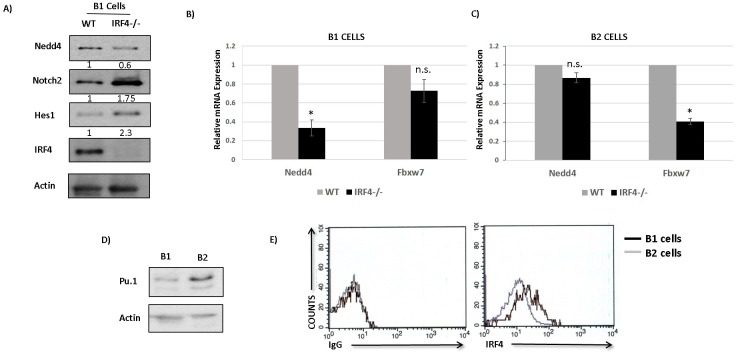
IRF4 regulates Nedd4 expression in B1 but not B2 cells **A.** Western-blot showing the levels of Nedd4, Notch2, Hes1 and IRF4 in IRF4^−/−^ B1 cells compared to IRF4^+/+^ B1 cells. The numbers below represent normalized relative expression calculated by densitometric quantification of respective proteins. **B.** Bar graph showing the relative mRNA expression of Nedd4 and Fbxw7 in IRF4^−/−^ and IRF4^+/+^ B1 cells. **C.** Bar graph showing the relative mRNA expression of Nedd4 and Fbxw7 in IRF4^−/−^ and IRF4^+/+^ B2 cells. **D.** Western-blot analysis to detect the levels of Pu.1 in B1 cells isolated from PC and B2 cells isolated from spleen of wild type mice. **E.** Flow cytometry analysis using intracellular staining to measure the levels of IRF4 in PC B1 cells and splenic B2 cells. The histograms represents intracellular staining with isotype control antibody (left panel) and with IRF4 antibody (right panel). Gray line represents B2 cells and Black line represents B1 cells. Cells were gated specifically on B1 and B2 populations based on IgM and B220 staining. *p value ≤0.01.

A previous study suggested that IRF4 may regulate the expression of Fbxw7 in B2 cells [19]. Our results show that IRF4 regulates Nedd4 but not Fbxw7 expression in CLL cells. It appears that expression of Nedd4 and Fbxw7 may be differentially regulated by IRF4 in different B cell subsets. To clarify this issue, we measured the expression of Fbxw7 and Nedd4 in B cell subsets isolated from IRF4 proficient and deficient mice. We first analyzed the levels of Nedd4 in IRF4 deficient B1 cells. Our result shows that IRF4 deficiency in B1 cells led to a significant decrease in expression of Nedd4 at the level of protein as well as mRNA (Figure 6A and 6B). The observed decrease in Nedd4 in IRF4^−/−^ B1 cells was accompanied by an increase in Notch2 expression and a corresponding increase in Hes1 (Figure 6A). However, compared to IRF4 proficient B1 cells we did not observe a significant change in the expression of Fbxw7 in IRF4 deficient B1 cells (Figure 6B). Surprisingly, IRF4 deficiency in splenic B2 cells was not associated with a significant change in the expression of Nedd4 (Figure 6C). Conversely, Fbxw7 levels were decreased in IRF4 deficient B2 cells (Figure 6C). These results confirm that expression of Nedd4 and Fbxw7 are differentially regulated by IRF4 in distinct B cell subsets.

IRF4 binds to DNA either as a homodimer or as a heterodimer with other transcription factors. It has been shown that DNA binding affinity of IRF4 for its target genes can be influenced by its own concentration as well as by the availability of its interacting partners [27]. Pu.1 belongs to Ets family of transcription factor and is a key interaction partner for IRF4 in B cells [27]. Therefore, to understand the observed discrepancy in the regulation of Nedd4 by IRF4 in B1 *versus* B2 cells, we assessed the levels of IRF4 and Pu.1. Intriguingly, the expression levels of Pu.1 were significantly higher in IRF4^+/+^ B2 cells than in IRF4^+/+^ B1 cells (Figure 6D). This finding is consistent with a previous report describing lower expression of Pu.1 mRNA in B1 cells [28]. The expression levels of Spi-b, which also belongs to the Ets family of transcription factors, were much lower and unaltered between B1 and B2 cells (data not shown). Interestingly, intracellular staining analysis further reveals that IRF4 was expressed at much higher levels in B1 cells than in B2 cells (Figure 6E). Collectively, these results show that IRF4 directly binds to *nedd4* gene locus to regulate its expression in B1 cells but not B2 cells and that expression levels of IRF4 and Pu.1 are distinct in B1 and B2 cells.

### IRF4 regulates Nedd4 expression in human B cells and CLL cells to downregulate Notch protein

**Figure 7:**
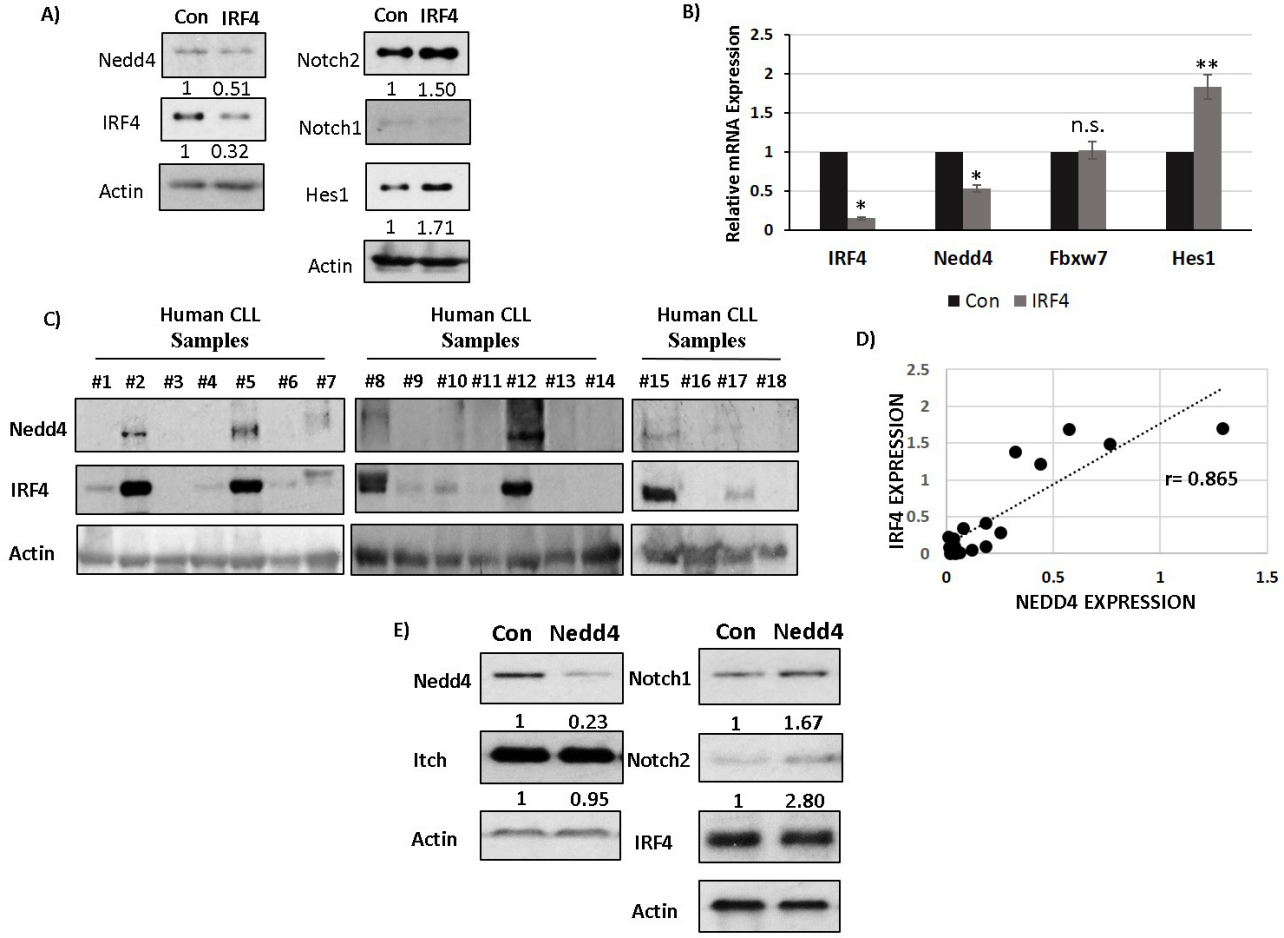
IRF4 regulates Nedd4 expression in human B cells and CLL cells to downregulate Notch protein **A.** Western-blot showing Nedd4, IRF4, Notch2, Notch1, Hes1 and Actin expression upon IRF4 knockdown using an IRF4 specific (IRF4) or scrambled control (Con) siRNA in normal human B cells isolated from healthy donors. The numbers below represents normalized relative expression. **B.** Bar graph showing relative mRNA expression of IRF4, Nedd4, Hes1 and Fbxw7 in normal human B cells in control *versus* IRF4 specific siRNA. **C.** Western blot analysis showing Nedd4 and IRF4 expression in human CLL samples represented by each individual lane. **D.** Scatter plot to show the correlation between IRF4 and Nedd4 protein expression in human CLL cells. The dotted line represents the linear trend line. Pearson correlation coefficient (r) value is 0.865. **E.** Western-blot analysis of Nedd4, Itch, Notch1, Notch2 and IRF4 following Nedd4 knockdown using siRNA in human Mec-1 CLL cells. Knockdown with scrambled siRNA is used as controls (con). The numbers below represent the normalized relative expression of respective genes measured by densitometric analysis. (PBMCs).*p value ≤0.0001 **p value ≤0.01.

We next wanted to determine whether IRF4 regulates Nedd4 expression in human B cells. To study this, we manipulated the levels of IRF4 using siRNA mediated knockdown in normal human B cells. Normal human B cells were nucleofected with a pool of siRNAs specific to IRF4 mRNA and with a pool of scrambled siRNAs as control (Figure 7A). siRNAs specific to IRF4 led to a strong decrease in the expression of IRF4 and a corresponding increase in the protein levels of Notch2 in normal human B cells (Figure 7A and 7B). Similar to murine B cells the expression levels of Notch1 protein was much lower compared to Notch2 (Figure 7A). Further mRNA and protein analysis showed a decrease in the expression of Nedd4 and a concurrent increase of Hes1 (Figure 7A and 7B). Importantly, the levels of Fbxw7 remained unaffected by IRF4 knockdown in normal human B cells (Figure 7B). We then assessed the protein levels of IRF4 and Nedd4 in primary human CLL cells. The levels of IRF4 were mostly low in human CLL samples (Figure 7C). However, some of the CLL samples particularly those with good prognosis (based on CD38 negativity) expressed IRF4 (Figure 7C and Supplementary Table T1). Interestingly, CLL samples expressing IRF4 also showed detectable Nedd4 expression (Figure 7C). We observed a high degree of correlation between IRF4 and Nedd4 expression among CLL samples (Figure 7D). In conclusion, these studies establish the conservation of IRF4 and Nedd4 regulatory axis in human B cells and CLL cells.

Effect of Nedd4 on Notch protein turnover has been mainly studied in drosophila. Here, we wanted to further determine whether Nedd4 regulates Notch protein turnover in CLL cells. We used a siRNA mediated knockdown approach to manipulate Nedd4 protein levels in human Mec-1 CLL cells. Mec-1 cells are an established human CLL cell line that expresses both Notch1 and Notch2 proteins. Knockdown of Nedd4 for 48 hours in Mec-1 cells led to an increase in the expression of both Notch1 and Notch2 proteins compared to knockdown with scrambled siRNA controls (Figure 7E). Importantly, the protein levels of Itch as well as IRF4 remained unaffected by Nedd4 protein knockdown (Figure 7E). Therefore, we conclude that Nedd4 can regulate Notch proteins turnover in CLL cells.

## DISCUSSION

Genetic evidence point towards an important role for Notch signaling in the pathogenesis of CLL [2, 5]. However, the significance of Notch signaling in the development of CLL *in vivo* has not been examined. Our studies here provide the first *in vivo* genetic evidence that Notch signaling is essential for development of CLL. Our findings support a role of Notch signaling in CLL initiation. This conclusion is supported by our results showing a significant delay in the onset of CLL upon *notch2* gene deletion. Our studies also reveal an absolute requirement of *notch2* gene for the generation of CLL cells in CD19creNotch2^fl/fl^IRF4^−/−^Vh11 mice. Moreover, our results show that Notch signaling promoted the survival and proliferation of CLL precursors (B1 cells) which may directly contribute to CLL initiation *in vivo*. A role for Notch in CLL initiation is further supported by a recent genomic analysis which shows that Notch mutations can be detected in early hematopoietic progenitor cells of CLL patients [29]. The frequency of Notch mutations are dramatically increased in therapy-resistant CLL patients, indicating a role of Notch in disease progression [1, 2]. Intriguingly, we also observed a detrimental effect on CLL cells survival and proliferation when *notch2* gene was deleted in the IRF4^−/−^Vh11 mice after onset of CLL with an inducible cre (data not shown). This result would indicate that Notch signaling is also important for CLL maintenance.

Constitutive activation of Notch signaling is reported in patients without Notch mutations [8, 30]. However, the molecular mechanisms leading to aberrant Notch signaling in CLL cells remain poorly defined. Our results presented here establish IRF4 as a critical regulator of Notch signaling during CLL development. Since, low levels of IRF4 is a common feature of CLL, the deregulated IRF4-Notch axis may represent a major pathway in the molecular pathogenesis of CLL. Interestingly, we identify Nedd4 as a key IRF4 target gene involved in impeding the responses of CLL cells and their precursors to Notch signaling. Intriguingly, a recent GWA study identified SNPs upstream to the *nedd4* gene locus to be strongly associated with CLL development in human patients [31]. Although, the functional significance of these SNPs on Nedd4 expression remains to be determined, our *in silico* analysis using a large cohort of CLL samples showed a significant decrease in Nedd4 expression in CLL cells compared to normal peripheral blood mononuclear cells (Figure S7). As an E3 ubiquitin ligase, Nedd4 may have many targets in CLL cells; however, our findings would indicate that Notch proteins are major targets of Nedd4 in the context of CLL development.

Our studies show that IRF4 regulates expression of Nedd4 in B1 cells but not B2 cells. This apparent paradoxical findings, we believe, can be explained by a recently proposed “kinetic control” model [27]. According to this model, the DNA binding landscape of IRF4 is influenced by the levels of IRF4 expression and the expression of its various interaction partners [27]. IRF4 can hetero-dimerize with Ets family of transcription factors to bind Ets-IRF Composite Elements (EICE) (GGAANNGAAA), while upon homo-dimerization IRF4 binds to ISRE motifs (GAAANNGAAA). Notably, IRF4-Ets heterodimers binds EICE motifs with much higher affinity compared to the binding of IRF4 homodimers to ISRE sites [27]. Moreover, this model may imply that binding of IRF4 homodimers to ISRE motifs may not occur efficiently in the presence of Ets transcription factors like Pu.1. Our results show that IRF4 is expressed at much higher levels in B1 than B2 cells. In contrast, Pu.1 is expressed at much higher levels in B2 cells than B1 cells. Therefore, the high levels of IRF4 and low levels of Pu.1 would lead to preferential binding of IRF4 to ISRE motifs present in *nedd4* gene promoter in B1 cells. On the other hand, higher levels of Pu.1 and lower levels of IRF4 in B2 cells may sequester IRF4 to EICE motifs and away from the low affinity ISRE motifs thereby, preventing its binding to *nedd4* gene promoter in B2 cells. Unlike Nedd4, the Fbxw7 expression was not significantly affected in IRF4^−/−^Vh11 B1 and CLL cells. These results indicate that Fbxw7 is not the major E3 ubiquitin ligase responsible for increased Notch receptor expression and signaling in IRF4 deficient B1 and CLL cells. It appears that Fbxw7, not Nedd4, is the E3 ubiquitin ligase that controls Notch activity in B2 cells. More studies are needed to determine whether Fbxw7 is a direct target of IRF4 that regulates Notch turnover in B2 cells.

In summary, our studies presented here uncover a novel regulatory pathway that controls Notch activity and CLL development. The importance of this pathway is strongly supported by the evidence that components of this pathway IRF4, Nedd4 and Notch are themselves frequently targeted during CLL development and progression [2, 5, 13, 31]. Therefore, deregulation of this pathway may represent a major pathogenesis step during CLL development and progression. Identification of this novel regulatory pathway not only helps us better understand the biology of CLL but could also offer new targets for diagnosis and therapeutic intervention.

## MATERIALS AND METHODS

### Animal studies

IRF4^−/−^Vh11 mice were generated and monitored for CLL development as previously described [15]. Notch2 floxed [32], Rosa-rtTA [33] and CD19cre [34] mice were generated as described previously and purchased from Jackson laboratory. NOD-scid gamma chain deficient mice were obtained from Jackson laboratory. TRE-IRF4 transgenic mice were generated and treated with doxycycline as previously described [35]. All animal studies were conducted on C57B6/129S mouse genetic background. All experiments were performed according to the guidelines from National Institute of Health and with an approved protocol from Institutional Animal Care and Use Committee of the University of Nebraska Medical Center.

### Human studies

All the human samples were collected and processed according to an approved protocol from Institutional Review Board. An informed written consent was obtained from each participant. The cells were isolated as previously described [36].

### Flow cytometry and cell sorting

The cells were isolated from respective tissues and pre-incubated with Fc-Block antibody. Antibodies against mouse B220, IgM and CD5 proteins were purchased from BD-pharmingen. Anti-mouse Notch2 and the corresponding isotype control antibodies were purchased from Biolegend. The anti-IRF4 antibody and the corresponding control antibody for intracellular staining were purchased from ebioscience. Fluorescence activated cell sorter (FACS) analysis was performed using FACSCalibur flow cytometer. Cell sorting was performed using BD FACSAria flow cytometer.

### Primary and cell cultures

All primary cells and Mec-1 CLL cell line were cultured in RPMI-1640 media containing 10% fetal bovine serum, 50μM Beta mercaptoethanol, 2mM L-glutamine and 100 U of penicillin and streptomycin. The B1 cells were isolated from peritoneal ascites following incubation in the tissue culture dishes for 6 hours to remove adherent macrophages.

### Calculation of Notch2 deletion efficiency

Genomic DNA was isolated from respective sort purified fractions by using Flexigene50 DNA extraction kit from Qiagen. The extracted DNA was subjected to real-time PCR using specific primers. Notch2 deletion efficiency was calculated by specifically designing PCR primers in region within the Notch2 conditional allele that is flanked by the loxP sites. This approach allowed PCR amplification of Notch2 alleles that have not undergone cre mediated deletion. Furthermore, we also amplified a non-related region in the genome and used it as control to normalize the result. The primer sequences used are included in Supplementary Table ST2.

### Western blotting

B-cells and CLL-cells from spleen were isolated by negative selection using MACS columns. Lysates were prepared and resolved using SDS-PAGE. The membranes were incubated with the indicated antibodies and Horse radish peroxidase (HRP) conjugated secondary antibodies. The signals were generated using Enhanced Chemi-Luminescence (ECL) substrate solution from Thermo-Pierce. The antibodies against Notch1, Notch2, Itch, Nedd4 and Hes1 were purchased from Cell signaling Technologies. Antibodies against IRF4 and Pu.1 were obtained from Santa Cruz Biotechnology. Direct HRP conjugated antibody against Beta Actin was purchased from Sigma. Nuclear extraction was performed using Nuclear and cytoplasmic extraction kit from Thermo scientific (Catalog #78833).

### Proliferation and survival assays

For Bromodeoxyuridine (BrDU) incorporation assay, cells were incubated in 10μM BrDU for 90 minutes to allow incorporation. BrDU positive cells were later detected using an Anti-BrDU staining kit from BD-pharmingen according to the manufacturer's instruction. Carboxyfluoroscein succinimidyl ester (CFSE) dye was purchased from Invitrogen to measure cell proliferation according to manufacturer's instructions. Apoptotic cells were detected using an AnnexinV staining kit from BD pharmingen.

### Transfection of Mec-1 CLL and normal B cells

Mec-1 cells were transfected using the Solution V kit purchased from Lonza. The transfection were carried out in a Nucleofector (Lonza) using the program X-001. Normal human B-cells were isolated from the peripheral blood of healthy donors using MACS magnetic beads separation. Transfections of normal human B cells were carried out in a Nucleofector (normal human B cell solution) using the program U-015. The siRNA against human IRF4 (on-target plus smart pool) were purchased from Dharmacon (L-019668-00-0005). The siRNA against human Nedd4 (on-target plus smart pool) were purchased from Dharmacon (L-007178-00-0005). The ON-target plus Non-targeting siRNA purchased from Dharmacon were used as controls (D001810-10-05). The cells were analyzed 48 hours post transfections.

### CLL transplantation

Whole splenocytes were isolated from mice with overt CLL. CLL was transplanted by intraperitoneal (IP) injections of 10^7^ whole splenocytes into the sublethally irradiated (2 grays) NSG mice.

### Statistical analysis

Each experiment was repeated at least three times unless otherwise indicated. The data in the bar graphs are represented with ± standard deviation. Two-tailed Student *t*-test was used to calculate p values to determine the significance. p value below 0.05 is considered statistically significant. Kaplan Meier survival analysis was performed using the log-rank test. The correlation between IRF4 and Nedd4 expression in human CLL cells was calculated using Pearson correlation coefficient, r.

## SUPPLEMENTARY MATERIALS FIGURES AND TABLES


