# Supplementary material for: Interferon regulatory factor 4 attenuates Notch signaling to suppress the development of chronic lymphocytic leukemia
Source: Oncotarget. 2016 May 25;7(27):41081–94. doi: 10.18632/oncotarget.9596 (PMC5173044; doi:10.18632/oncotarget.9596)
Supplement: Supplementary file 1 [file oncotarget-07-41081-s001.pdf]

Interferon regulatory factor 4 attenuates notch signaling to suppress the development of chronic lymphocytic leukemia

Supplementary Material

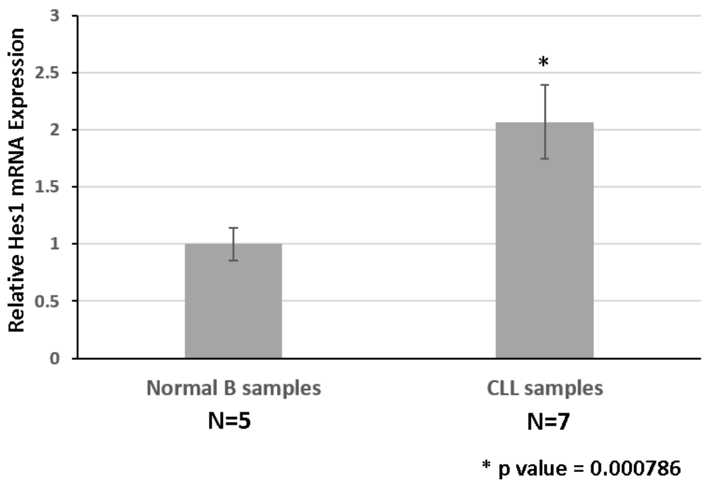

Figure S1 **Hes1 mRNA expression in primary human CLL cells**. Bar graph showing the mRNA expression of Hes1 in primary human CLL cells (N=7) compared to normal human B cells (N=5).

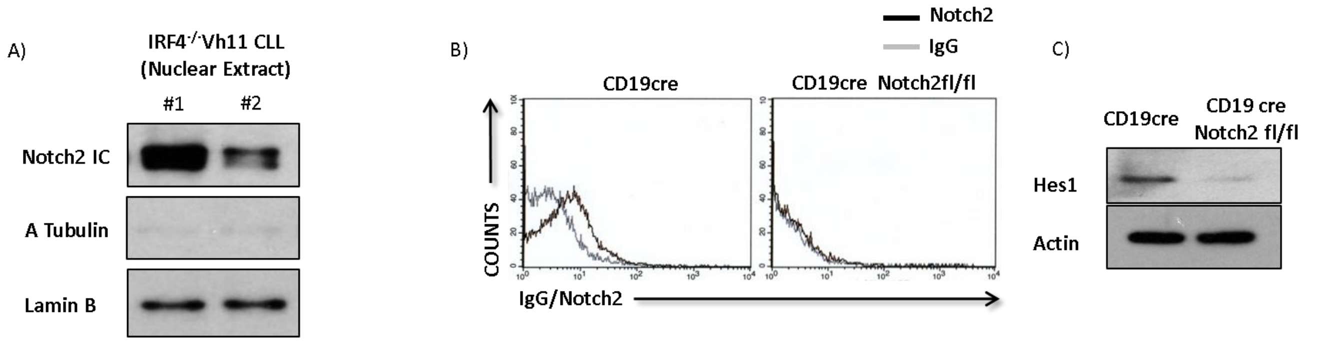

FigureS2 Notch2 protein is critical for Notch signaling in B cell *in vivo*. (A) Nuclear fractionation of IRF4$^{-/-}$Vh11 CLL cells to detect the intracellular domain of Notch2. Lamin B is used as a positive loading control and alpha tubulin (A tubulin) is used as a negative loading control. (B) Flow cytometry staining for IgG and Notch2 represented as histograms. Gray line represents IgG staining and black line represents Notch2 staining in B-cells. Left panel indicates CD19cre control mouse and right panel shows CD19cre Notch2$^{fl/fl}$ mouse. (C) Western-blots showing Hes1 protein in B-cells isolated from CD19cre and CD19cre Notch2$^{fl/fl}$ mice.

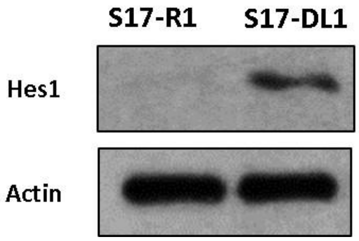

Figure S3 **Hes1 induction upon co-culture with S17-DL1 stromal-cells**. Western Blot analysis to measure Hes1 levels in B1-cells co-cultured with S17-R1 and S17-DL-1 stromal-cells for 48 hours.

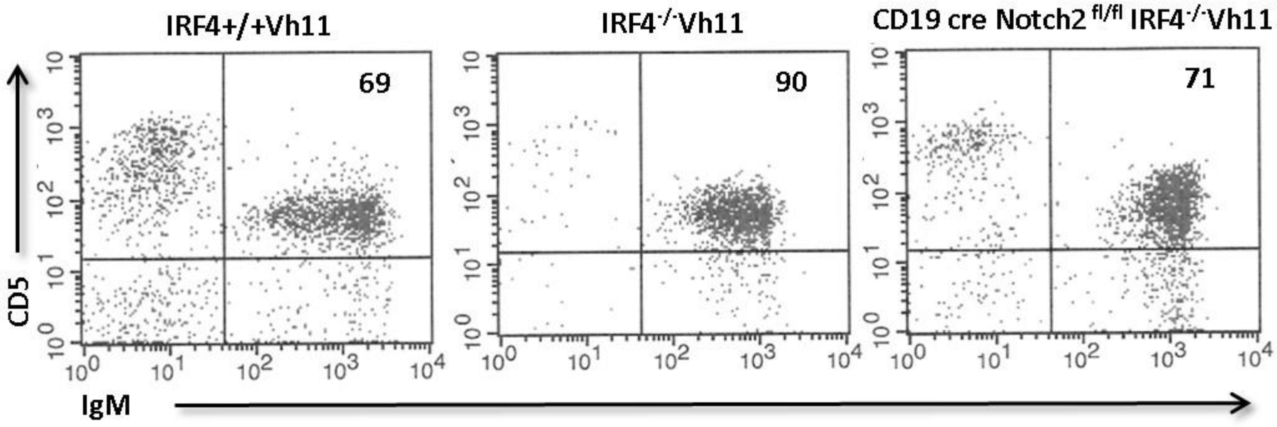

Figure S4 Normal B1 cell generation in CD19creNotch2$^{fl/fl}$IRF4$^{-/-}$Vh11 mice. Flow cytometry staining to detect IgM+ CD5+ B1-cells in peritoneal cavities of IRF4$^{+/+}$Vh11, IRF4$^{-/-}$Vh11 and CD19creNotch2$^{fl/fl}$IRF4$^{-/-}$Vh11 (no CLL) mice. The numbers represents the frequency of B1-cells.

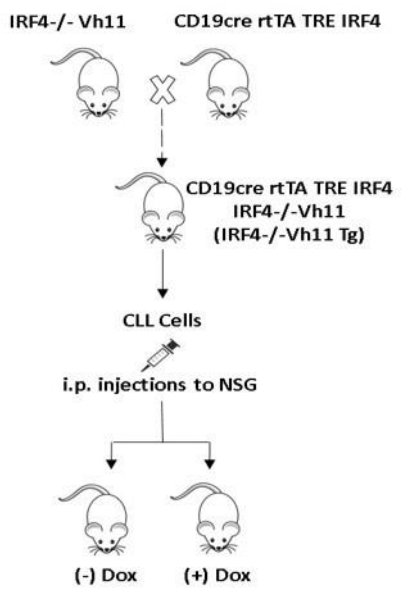

Figure S5 Schematic showing the breeding scheme and experimental design for IRF4 reconstitution in IRF4$^{-/-}$ Vh11 CLL-cells. To accomplish this, we used a previously described tetracycline response element driven IRF4 transgenic line (TRE-IRF4)[1]. A transgenic mouse expressing a cre-recombinase activated reverse tetracycline-controlled transactivator with an EGFP reporter (rtTA) was used to drive the expression of IRF4 transgene *in vivo*. We bred mice containing rtTA and TRE-IRF4 alleles to CD19cre mice to generate CD19cre rtTA TRE-IRF4 mice which upon treatment with doxycycline (Dox), allow IRF4 transgene expression exclusively in B-cells. Through further breeding we introduced these alleles (CD19cre rtTA TRE-IRF4) into the IRF4$^{-/-}$Vh11 mice to generate CD19cre rtTA TRE-IRF4 IRF4$^{-/-}$Vh11 mice (IRF4$^{-/-}$Vh11Tg). The blood was analyzed regularly from IRF4$^{-/-}$ Vh11Tg mice. Upon CLL development in IRF4$^{-/-}$Vh11Tg mice, the CLL-cells were isolated from spleen and transplanted to NOD-scid gamma deficient (NSG) immunocompromised mice. The blood from the NSG mice was screened for the development of overt CLL. Upon successful establishment of CLL the NSG mice, were fed either with dox containing water to induce IRF4 expression (NSG(+)Dox) or with water without dox to be used as controls (NSG(-)Dox).

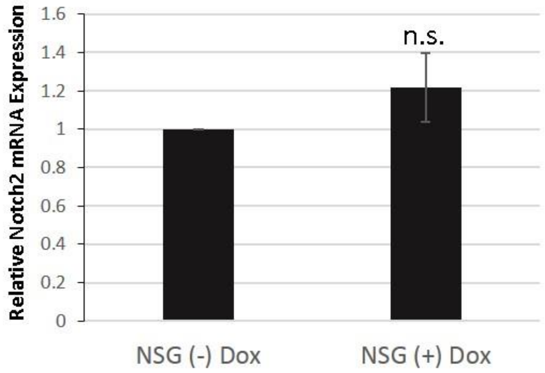

Figure S6 **Notch2 mRNA expression upon IRF4 reconstitution.** Bar graph showing relative mRNA expression of Notch2 in CLL-cells isolated from NSG mice fed with or without dox water.

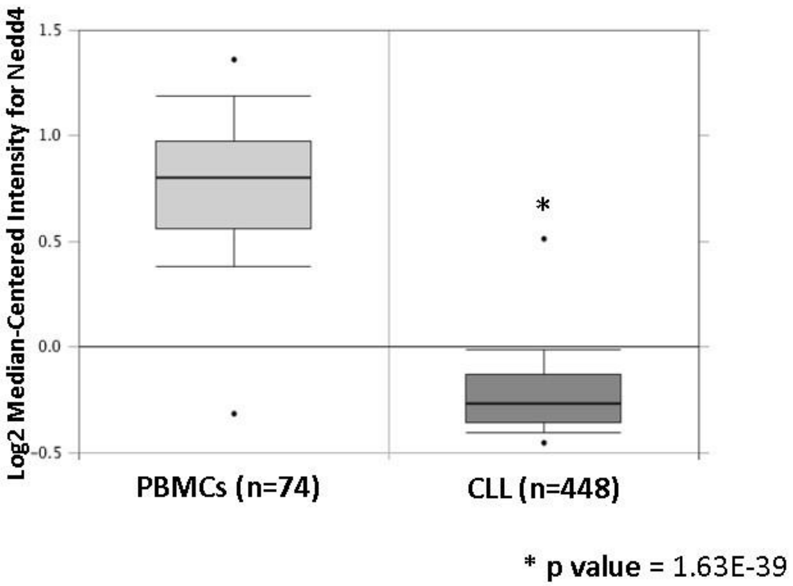

Figure S7 **Nedd4 mRNA expression in human CLL patients.** Box plot showing the Nedd4 mRNA expression in a large cohort of CLL samples compared to normal peripheral blood mononuclear cells (PBMCs). The dataset used for analysis was Haferlach leukemia dataset from oncomine.

Supplementary Table T1

CLL Patient #	Source	Cytogenetics	IgVH mutation status	CD38 positivity	Monotypic B-cells
CLL 1	PB	Trisomy 12	Unmutated	Pos	93%
CLL 2	PB	11q23, 13q14 del	Unmutated	Neg	95%
CLL 3	PB	11q23	Not done	Pos	74%
CLL 4	PB	17p del, 13q	Unmutated	Pos	92%
CLL 5	PB	Normal	Mutated	Neg	82%
CLL 6	PB	13q14 del	Unmutated	Neg	61%
CLL 7	PB	13q14 del	Not done	Neg	53%
CLL 8	PB	13q14 del	Not done	Neg	74%
CLL 9	PB	Normal	Mutated	Pos	78%
CLL 10	PB	13q null	Not done	Neg	75%
CLL 11	PB	Normal	Not done	Pos	43%
CLL 12	PB	11q23, 13q14 del	Not done	Neg	93%
CLL 13	PB	Normal	Not done	Pos	85%
CLL 14	PB	Trisomy 12	Not done	Neg	77%
CLL 15	PB	Normal	Mutated	Neg	97%
CLL 16	PB	13q14 del	Not Done	Neg	80%
CLL 17	PB	13q14 del	Not Done	Neg	85%

CLL 18	PB	11q23, 13q14 del	Unmutated	Neg	67%

Supplementary Methods

Chromatin Immuno-precipitation (ChIP) Sequencing. B1-cells were isolated and fixed with 1% formaldehyde for 15 min and quenched with 0.125 M glycine. Chromatin was isolated by the addition of lysis buffer, followed by disruption with a Dounce homogenizer. Lysates were sonicated and the DNA sheared to an average length of 300-500 bp. Genomic DNA (Input) was prepared by treating aliquots of chromatin with RNase, proteinase K and heat for de-crosslinking, followed by ethanol precipitation. An aliquot of chromatin (30 ug) was precleared with protein G agarose beads (Invitrogen). Genomic DNA regions of interest were isolated using 4 ug of antibody against IRF4 (Santa Cruz, sc-6059,). Complexes were washed, eluted from the beads with SDS buffer, and subjected to RNase and proteinase K treatment. Crosslinks were reversed by incubation overnight at 65 C, and ChIP DNA was purified by phenol-chloroform extraction and ethanol precipitation.

Illumina sequencing libraries were prepared from the ChIP and Input DNAs by the standard consecutive enzymatic steps of end-polishing, dA-addition, and adaptor ligation. After a final PCR amplification step, the resulting DNA libraries were quantified and sequenced on Illumina's HiSeq 2500 (50 nt reads, single end). Reads were aligned to the mouse genome (mm10) using the BWA algorithm (default settings). Duplicate reads were removed and only uniquely mapped reads (mapping quality >= 25) were used for further analysis. Alignments were extended in silico at their 3'-ends to a length of 200 bp, which is the average genomic fragment length in the size-selected library, and assigned to 32-nt bins along the genome. The resulting histograms (genomic "signal maps") were stored in bigWig files. Peak locations were determined using the MACS algorithm (v1.4.2) with a cutoff of p-value = 1e-7. Signal maps and peak locations were used as input data to Active Motifs proprietary analysis program, which creates Excel tables containing detailed information on sample comparison,

peak metrics, peak locations and gene annotations. The representative data is generated by analyzing the data using the Integrated Genome Browser (IGB).

Real-time PCR. Cell lysis was performed using Trizol reagent. The total RNA was reverse transcribed using the first strand cDNA synthesis kit from GE healthcare. Real-time PCR was performed using SYBR-green reagent from Applied Biosystems in ABI 7500 cycler. Primers specific to the gene or region of interest were used for the levels of mRNA or CHIP assay respectively. A complete list of primers is included in the supplemental table 1 (Table ST1).

Supplementary Table ST2

**Primer**	**Forward**	**Reverse**
Notch2 Deletion	ATGTCCAGAGGGCTTCTTGGGAG	TGGCAGTCCTCTCCTGTGAATCC
Control Deletion	CTTCCTTTAGGGTAACTGGCCGCC	CAGGATAAAGGACACTCCACCCAG
Kappa CHIP	TAGCACAGAGTACCCACCCATATCTC	CTATCTTGGTCCATGGGACACTCCC
Nedd4 CHIP 2Kb	TGTTTCGGCTCATAATCTCATGGG	AACTGCACTACCACACCTGGCA
Nedd4 CHIP 4kb	CTGCCAGTGAAGCAGGAGCCTTTAG	GGGACCTAGAGTGGGCATCAAAA
human Nedd4 mRNA	ATGGCAACATTCAACTGCAA	GGCCTGGTTGCTATACATGG
human Fbxw7 mRNA	GACGCCGAATTACATCTGTC	GTAGCAGGTCTTTGGGTTC
human Hes1 mRNA	AGTGAAGCACCTCCGGAAC	TCACCTCGTTCATGCACTC
mouse Hes1 mRNA	GTGTCAACACGACACCGGACAAACC	GCTTGGAATGCCGGGAGCTATCT
mouse Notch2 mRNA	CAGCTCTAACCCGTGCCTGAATG	GGGCCTTGCCTTTTCCTGAACAC
mouse Nedd4 mRNA	TCACTGCTGATCCGTACCTGGAGC	GCTGGTAAGGATTCCACTCATCGGG
mouse Fbxw7 mRNA	TGCAAAGTCTCAGATTATACC	ACTTCTCTGGTCCGCTCCAGC

REFERENCES

1. Pathak S, Ma S, Trinh L, Eudy J, Wagner KU, Joshi SS and Lu R. IRF4 is a suppressor of c-Myc induced B cell leukemia. PloS one. 2011; 6(7):e22628.
